# Protocol for *in vitro* chemoenzymatic synthesis of thiazole-containing macrocyclic peptides via ribosomal incorporation of thioamides

**DOI:** 10.1016/j.xpro.2026.104762

**Published:** 2026-08-04

**Authors:** Akihiro Saito, Yuki Goto

**Affiliations:** 1 Department of Chemistry, Graduate School of Science, Kyoto University, Sakyo, Kyoto 606-8502, Japan; 2 Toyota Riken Rising Fellow, Toyota Physical and Chemical Research Institute, Sakyo, Kyoto 606-8502, Japan

**Keywords:** Protein expression and purification, Biotechnology and bioengineering, Chemistry

## Abstract

Backbone thiazole moieties (Thz) are prevalent structural features in peptidic natural products and contribute to favorable physicochemical properties. Here, we describe a protocol for *in vitro* chemoenzymatic synthesis of artificial Thz-containing macrocyclic peptides compatible with mRNA display workflows. We provide steps for preparing amino(thio)acid substrates and flexizyme-mediated tRNA acylation. We then detail procedures for ribosomal incorporation of thioamide linkages, spontaneous thioether macrocyclization and concomitant backbone thiazoline (Thn) formation, and enzymatic dehydrogenation of Thn to Thz to afford Thz-containing macrocycles.

For complete details on the use and execution of this protocol, please refer to Saito et al.^1^

## Highlights

• Protocol for chemoenzymatic synthesis of thiazole-containing macrocyclic peptides
• Steps for amino(thio)acid substrate synthesis for flexizyme-mediated tRNA acylation
• Guidance on ribosomal incorporation of thioamides and spontaneous thiazoline formation
• Instructions for enzymatic dehydrogenation of thiazolines to generate backbone thiazoles

## Abstract (editor highlights)

Publisher’s note: Undertaking any experimental protocol requires adherence to local institutional guidelines for laboratory safety and ethics.

## Graphical abstract

**Figure:**
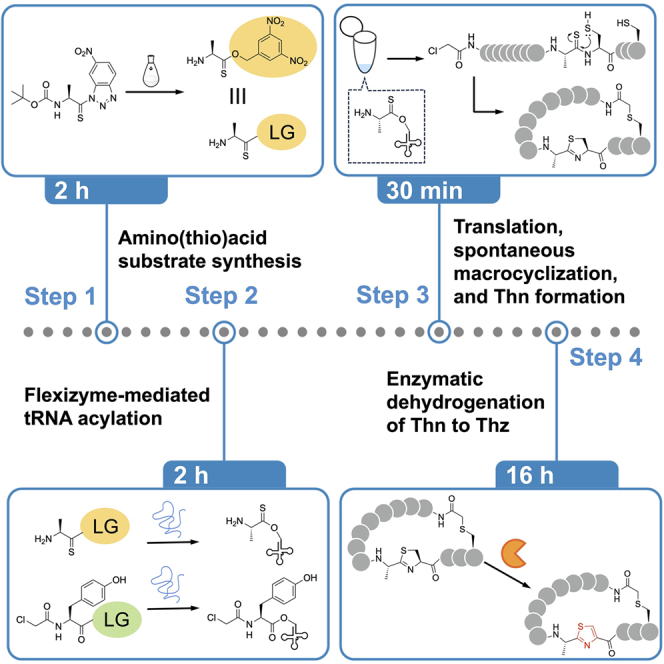

## Before you begin

Backbone thiazole moieties (Thz) are prevalent building blocks in peptidic natural products. These structures contribute to structural rigidity and unique hydrogen-bonding patterns, and these features are expected to improve membrane permeability and oral bioavailability of cyclic peptides relative to fully amidic peptides.^2^^,^^3^ Therefore, incorporating Thz moieties into synthetic peptide libraries is an attractive strategy for the de novo discovery of peptide ligands with improved physicochemical properties.

For ligand discovery, *in vitro* translation systems provide a powerful platform for constructing large combinatorial peptide libraries. However, existing protocols for posttranslational installation of Thz moieties into peptides typically require enzyme recognition sequences,^4^^,^^5^^,^^6^^,^^7^^,^^8^ or harsh chemical conditions,^9^ which restrict sequence diversity and limit compatibility with display-based screening methods. Also, direct ribosomal incorporation of Thz units into peptides requires engineered ribosomes, and the translation efficiency is generally low for practical applications.^10^^,^^11^ Thus, a mild aqueous synthetic approach applicable to a wide range of sequences would expand the applicability of azole-containing peptides in *in vitro* selection technologies.

**Figure 1:**
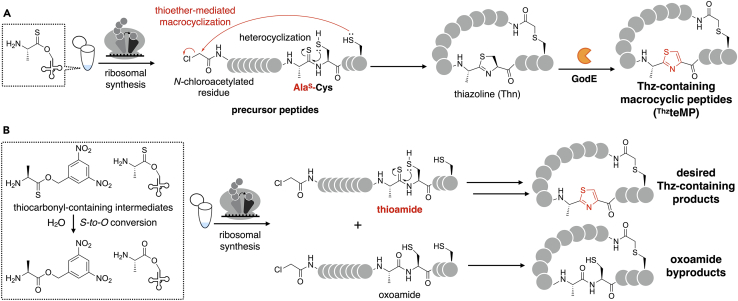
Schematic representation of the chemoenzymatic synthesis of ^Thz^teMP (A) Synthetic scheme for the desired ^Thz^teMP via ribosomal incorporation of Ala^S^. (B) Expected oxoamide byproduct formed through S-to-O conversion of Ala^S^-tRNA during synthesis.

Here, we describe a protocol for the chemoenzymatic synthesis of Thz-containing peptides through a sequence of reactions including flexizyme-mediated aminoacylation,^12^ ribosomal synthesis of thionoalanine (Ala^S^)-containing precursor peptides,^13^ spontaneous heterocyclization to form thiazoline (Thn), and enzymatic dehydrogenation catalyzed by the azoline dehydrogenase GodE to generate Thz (Figure 1A).^14^^,^^15^ By combining this method with the initiator codon reprogramming to install *N*-chloroacetylated residues for thioether-mediated macrocyclization, Thz-containing macrocyclic peptides (^Thz^teMPs) can be synthesized (Figure 1A). A key feature of this approach is the use of noncanonical building blocks that generate thioamide-containing intermediates prior to cyclization. Because thiocarbonyl-containing intermediates inevitably undergo *S-to-O* conversion in aqueous environments,^13^ this synthetic scheme inherently produces mixtures of the desired Thz-containing products and corresponding oxoamide byproducts (Figure 1B). In the protocol presented here, careful reaction setup and optimized reaction conditions successfully minimize the formation of these byproducts. The protocol below describes the specific workflow used for incorporating thioamide precursors into peptides during *in vitro* translation and converting them into ^Thz^teMPs suitable for downstream mRNA display-based selection experiments.

### Innovation

Our synthetic method utilizes the unique reactivity of thioamides, enabling mild posttranslational conversion without strict requirements for enzyme substrate recognition. These features are critical for the construction and screening of highly diverse peptide libraries using the mRNA display method.^16^^,^^17^ In addition to its application to display-based peptide ligand discovery, this protocol also enables the preparation of structurally constrained, natural-product-like peptides for biochemical or chemical biology studies.

## Key resources table

**Table:** 

REAGENT or RESOURCE	SOURCE	IDENTIFIER
**Chemicals, peptides, and recombinant proteins**		

Boc-thionoala-1-(6-nitro)benzotriazolide	Watanabe	Cat#M01832; CAS: 184951-86-8
3,5-Dinitorobenzyl alcohol	Angene	Cat#AG0061J2; CAS: 71022-43-0
*N,N*-Diisopropylethylamine (DIPEA)	Tokyo Chemical Industry	Cat#D1599; CAS: 7087-68-5
Dichloromethane (DCM), anhydrous	Wako	Cat#044-31235; CAS: 75-09-2
Hydrochloric acid (HCl)	Wako	Cat#080-01066; CAS: 7647-01-0
Sodium hydrogen carbonate (NaHCO_3_)	Wako	Cat#191-01305; CAS: 144-55-8
Sodium sulfate (Na_2_SO_4_)	Wako	Cat#197-03345; CAS: 7757-82-6
Hexane	Wako	Cat#083-00417; CAS: 110-54-3
Ethyl acetate (EtOAc)	Wako	Cat#059-00357; CAS: 141-78-6
4 N HCl in EtOAc	Watanabe	Cat#A00024
1-Methyl-2-pyrrolidone (NMP)	Nacalai tesque	Cat#23030-55; CAS: 872-50-4
Dimethyl sulfoxide (DMSO)	Nacalai tesque	Cat#13407-45; CAS: 67-68-5
*N*-(2-Hydroxyethyl)piperazine-*N*′-2-ethanesulfonic acid (HEPES)	Nacalai tesque	Cat#17546-05; CAS: 7365-45-9
Potassium hydroxide (KOH)	Nacalai tesque	Cat#28617-35; CAS: 1310-58-3
Magnesium chloride hexahydrate (MgCl_2_)	Nacalai tesque	Cat#20909-55; CAS: 7791-18-6
3 M Sodium acetate (NaOAc) buffer solution	Nacalai tesque	Cat#31138-31
Ethanol	Nacalai tesque	Cat#14711-15; CAS: 64-17-5
Methanol (MeOH), LC-MS grade	Wako	Cat#138-14521; CAS: 67-56-1
Acetic acid (AcOH), LC-MS grade	Wako	Cat#018-20061; CAS: 64-19-7
Acetonitrile (MeCN), LC-MS grade	Wako	Cat#018-19853; CAS: 75-05-8
Recombinant GodE protein	Onaka et al.^14^ Ozaki et al.^15^	N/A
10× TS solution	Goto et al.^18^	N/A
10× RP solution	Goto et al.^18^	N/A
10× ARS solution	Goto et al.^18^	N/A

**Oligonucleotides**		

Primers for eFx, dFx see Table 1	This paper	N/A
Primers for tRNA^EnAsn#3^_cua_, tRNA^fMet^_cua_, see Table 1	This paper	N/A
Primers for DNA templates, see Table 2	This paper	N/A

**Table 1:** Primers used for the preparation of flexizymes and tRNAs

Primer	Sequence (5′→ 3′)
T7eSD6M.F46	TAATACGACTCACTATAGGGTTAACTTTAACAAGGAGAAAAACATG
T7ex5.F22	GGCGTAATACGACTCACTATAG
Fx5'.F36	GTAATACGACTCACTATAGGATCGAAAGATTTCCGC
eFx.R45	ACCTAACGCTAATCCCCTTTCGGGGCCGCGGAAATCTTTCGATCC
eFx.R18	ACCTAACGCTAATCCCCT
dFx.R46	GGATCGAAAGATTTCCGCATCCCCGAAAGGGTACATGGCGTTAGGT
dFx.R19	ACCTAACGCCATGTACCCT
Ini1-1G-5'.F49	GTAATACGACTCACTATAGGCGGGGTGGAGCAGCCTGGTAGCTCGTCGG
Ini cat.R44	GAACCGACGATCTTCGGGTTATGAGCCCGACGAGCTACCAGGCT
Ini-3'.R38	TGGTTGCGGGGGCCGGATTTGAACCGACGATCTTCGGG
Ini-3'.R20	TG(Me)GTTGCGGGGGCCGGATTT
EnAsn-5'.F49	GTAATACGACTCACTATAGGCTCTGTAGTTCAGTCGGTAGAACGGCGG
EnNcau#3.R58	GCGGCTCTGAGGGGACTCGAACCCCTGACATACGGATTATGAGTCCGCCG TTCTACCG
EnN3′(AGGG)OMe.R16	TG(Me)GCGGCTCTGAGGGG

**Table 2:** Primers used for the preparation of DNA templates

Primer	Sequence (5′→ 3′)
pripep1.R49	GCAACCAGCTCCGCACATCCCACCTGCGGCTTTTTTCATGTTTTTCTCC
pripep1.R18	AGCTTAGCAACCAGCTCC
pripep2.R59	AGGAGAAAAACATGGCGGGGTTGGAGGCAGCTATGTGCCTGGGTGGCTATTTACGCTGC
pripep2.R19	CTATTTACGCTGCTAAGCT

## Materials and equipment

• Flexizymes (250 μM eFx, 250 μM dFx), tRNAs (250 μM tRNA^EnAsn#3^_cua_,^19^ 250 μM tRNA^fMet^_cua_), DNA templates for translation

Prepare flexizymes, tRNAs, and DNA template encoding the target peptides by PCR and *in vitro* transcription following the previously published protocols.^12^^,^^18^^,^^20^ Use the primers listed in Table 1 for each flexizyme and each tRNA preparation. Use the primers listed in Table 2 to prepare the DNA template for the model sequence used in this protocol. Combinations of the primers for PCR are listed in Table 3.

• ^ClAc^Tyr-CME in DMSO

**Table 3:** Primer combinations for each DNA template

	Primer extension		PCR	
	Forward	Reverse	Forward	Reverse
eFx	Fx5'.F36	eFx.R45	T7ex5.F22	eFx.R18
dFx	Fx5'.F36	dFx.R46	T7ex5.F22	dFx.R19
tRNA^fMet^_cua_	Ini1-1G-5'.F49	Ini cat.R44	T7ex5.F22	Ini-3'.R38 (1st), Ini-3'.R20 (2nd)
tRNA^EnAsn#3^_cua_	EnAsn-5'.F49	EnNcau#3.R58	T7ex5.F22	EnN3′(AGGG)OMe.R16
pep1	T7eSD6M.F46	pripep1.R49	T7ex5.F22	pripep1.R18
pep2	T7eSD6M.F46	pripep2.R59	T7ex5.F22	pripep2.R19

Synthesize the cyanomethyl ester (CME)-activated substrate for flexizyme-mediated aminoacylation via a two-step synthesis from Tyr according to the previously reported method.^21^ Dissolve the resulting product in DMSO to yield a 200 mM stock, which typically remains stable for up to one year at −20°C. To prepare a working solution, dilute the 200 mM stock with DMSO to a final concentration of 25 mM. These aliquots can be stored at −20°C and are generally stable for at least one year.

• Enzyme solution (30 μM GodE)

Express GodE as a C-terminally His-tagged protein in *E. coli*. BL21(DE3) using the pET-26b(+) vector and purify it by Ni-NTA affinity chromatography.^15^ Aliquot the resulting enzyme solution into small volumes to avoid repeated freeze–thaw cycles. These aliquots can be stored at −80°C and are generally stable for at least one year.

• 5 mM each amino acid solution (−M)

Mix proteinogenic amino acids except for Met to prepare a stock solution containing each amino acid at 5 mM.^18^ The solution can be stored at −20°C and is generally stable for at least one year.

• *In vitro* translation system (10× TS solution, 10× RP solution, 10× ARS solution)

Prepare the translation system by expressing the translation factors in *E. coli*. and purify them by Ni-NTA affinity chromatography or other appropriate methods. The purified proteins are then combined according to the published protocol.^18^ Aliquot the resulting translation system into small volumes to avoid repeated freeze–thaw cycles. These aliquots can be stored at −80°C and are generally stable for at least six months.

***Alternatives:*** Commercially available *in vitro* translation systems are also applicable. For codon reprogramming, the PURExpress® Δ (aa, tRNA) Kit (New England Biolabs, Cat#E6840S) is recommended to be used following the manufacturer’s instructions.

## Step-by-step method details

### Synthesis of amino(thio)acid substrate, Ala^S^-DNB

**Timing: 1 day**

**Figure 2:**
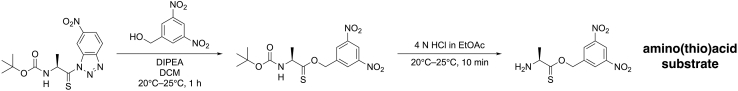
Synthetic scheme of Ala^S^-DNB

In this procedure, the amino(thio)acid substrate recognized by flexizyme is synthesized by a substitution reaction (Figure 2). The resulting amino(thio)acid substrate contains a 3,5-dinitrobenzyl (DNB) leaving group.

1. Place 351 mg of Boc-thionoala-1-(6-nitro)benzotriazolide (1.0 mmol, 1.0 eq.) and 238 mg of 3,5-dinitrobenzyl alcohol (1.2 mmol, 1.2 eq.) in an Erlenmeyer flask and dissolve them in 5 mL of anhydrous DCM.
2. Add 205 μL of DIPEA (1.2 mmol, 1.2 eq.) to the solution and stir at 20°C–25°C for 1 h. Monitor the reaction by TLC (Rf = 0.4, 4:1 hexane/EtOAc), visualizing the spots under UV light or with ninhydrin staining.
3. After the completion of the reaction, add 5 mL of 0.5 M HCl to the solution and extract the aqueous layer with DCM.
4. Wash the organic layer with sat. NaHCO_3_, brine, and dry the organic layer with Na_2_SO_4_.
5. Evaporate the solvent under reduced pressure.
6. Purify the resulting crude product by flash column chromatography using 10 g prepacked silica gel column with a gradient of 5–40% EtOAc in hexane over 10 column volumes. Troubleshooting 1.
7. Obtain the product as a yellow oil (160 mg, 42% yield).

**Pause point:** The product can be stored at −20°C for at least six months.

8. Dissolve the resulting product in 1 mL of chloroform.
9. Add 1 mL of 4 N HCl in EtOAc, and stir at 20°C–25°C for 10 min.
10. Evaporate the solvent under reduced pressure with a base trap.
11. Co-evaporate with diethyl ether 4-5 times.
12. Resuspend the precipitate in ether, transfer it to 1.5 mL tubes, and remove the supernatant.
13. Dry the precipitate in air for over 30 min and then dry under vacuum for at least 2 h.
14. Dissolve the resulting solid in NMP to make a 25 mM solution. Troubleshooting 2.

**Pause point:** The NMP solution of the product can be stored at −80°C for at least several months.

**CRITICAL:** Ala^S^-DNB should be stored as an NMP solution at −80°C to minimize *S-to-O* conversion during storage, rather than as a DMSO solution typically used for amino acid substrates in flexizyme-mediated aminoacylation.

### Flexizyme-mediated aminoacylation to give Ala^S^-tRNA and ^ClAc^Tyr-tRNA

**Timing: 3 h**

In this procedure, aminoacylated tRNAs are prepared using flexizymes. Ala^S^ is used for heterocyclization with an adjacent Cys residue, and ^ClAc^Tyr is used for initiator codon reprogramming and thioether-mediated macrocyclization.

15. Aminoacylation reaction.
  a. Prepare the aminoacylation reaction mixture in a 0.6 mL tube according to the following table.
    i. Aminoacylation reaction mixture for Ala^S^-tRNA

**Table:** 

Reagent	Amount
RNase-free water	6 μL
500 mM HEPES-KOH buffer (pH 7.5)	2 μL
250 μM dFx	2 μL
250 μM tRNA^EnAsn#3^_cua_	2 μL
    ii. Aminoacylation reaction mixture for ^ClAc^Tyr-tRNA

**Table:** 

Reagent	Amount
RNase-free water	3 μL
500 mM HEPES-KOH buffer (pH 7.5)	1 μL
250 μM eFx	1 μL
250 μM tRNA^fMet^_cua_	1 μL
      ***Note:*** The suppressor tRNA for Ala^S^ incorporation should be based on the EnAsn#3 scaffold,^19^ and its anticodon sequence should be CAU for AUG codon reprogramming.
  b. Heat the samples at 95°C for 2 min.
  c. Incubate the tubes at 20°C–25°C for 5 min.
  d. Add 4 μL of 3 M MgCl_2_ to (i) and 2 μL of 3 M MgCl_2_ to (ii).
  e. Incubate the tubes at 20°C–25°C for 5 min.
  f. Place the tubes on ice and leave them for 10 min.
  g. Add 2 μL of 25 mM ^ClAc^Tyr-CME (DMSO solution) to (ii), and incubate the tubes on ice.
  h. After 1.5 h, add 4 μL of 25 mM Ala^S^-DNB (NMP solution) to (i).
  i. Incubate the tubes on ice for an additional 30 min.
16. Ethanol precipitation.
  a. Add 20 μL of (i) and 10 μL of (ii) to 120 μL of 0.3 M NaOAc prechilled on ice.
  b. Add 300 μL of ethanol.
  c. Centrifuge the mixture at 15,300 × *g* for 15 min at 25°C and discard the supernatant.
  d. Add 150 μL of 70% ethanol containing 0.1 M NaOAc.
  e. Centrifuge the mixture at 15,300 × *g* for 5 min at 25°C and discard the supernatant.
  f. Repeat d. and e. one more time.
  g. Add 10 μL of 70% ethanol and centrifuge the sample at 15,300 × *g* for 3 min at 25°C.
  h. Discard the supernatant and air-dry the pellet for 5 min at 20°C–25°C.
  i. After the pellet is completely dried, place the tubes on ice.

***Note:*** Because lower temperatures can promote undesired salt co-precipitation, the ethanol precipitation and subsequent washing steps should be performed at 25°C. As these steps are carried out under weak acidic conditions, both *S-to-O* conversion and hydrolysis of the aminoacyl-tRNA are expected to be minimized despite the elevated temperature.

**CRITICAL:** It is not recommended to store the aminoacyl-tRNA as a pellet due to the instability of Ala^S^-tRNA. Perform this procedure as quickly as possible and proceed immediately to the next step.

***Optional:*** Aminoacylation can be evaluated by acid-PAGE analysis using a microhelix RNA (mhRNA) as a tRNA analog. However, as previously reported,^13^ this method does not resolve Ala^S^-mhRNA from the background of *S-to-O*-converted Ala-mhRNA. Therefore, the acid-PAGE assay serves only to confirm that acylation has occurred, whereas the actual thioacylation efficiency must be assessed through the ribosomal translation.

### Posttranslational chemoenzymatic synthesis of ^Thz^teMPs

**Timing: 17–18 h**

In this procedure, the desired ^Thz^teMPs are synthesized via *in vitro* translation and subsequent posttranslational modifications, and the products are analyzed by LC-MS.

17. Prepare the translation reaction mixture in 0.6 mL low-protein-binding tubes.

**Table:** Translation mixture

Reagent	Amount
5 mM each amino acid solution (-M)	0.5 μL
0.8 μM DNA template	0.25 μL
10× TS solution	0.5 μL
10× RP solution	0.5 μL
10× ARS solution	0.5 μL
RNase-free water	1.75 μL

***Note:*** The codon assigned to Ala^S^ should be the AUG codon for efficient incorporation. DNA template should contain an AUG-UGU/C motif to encode Ala^S^ followed by Cys, which is required for spontaneous heterocyclization to form Thn.

***Optional:*** To monitor the stepwise conversion from Thn to Thz by LC-MS analysis of Thn-containing intermediates, double the volume of the translation mixture and add water to the half aliquot instead of GodE for a GodE(−) sample.

18. Dissolve the pellet of aminoacyl-tRNA prepared in the previous step in 1 μL of 1 mM NaOAc.
19. Add the aminoacyl-tRNA solution to the translation mixture.
20. Incubate the solution at 37°C for 30 min. Troubleshooting 3.
21. Add 1 μL of 30 μM GodE stock solution to the mixture.
22. Incubate the solution at 25°C for at least 16 h. Troubleshooting 4 and 5.
23. Add 6 μL of LC-MS-grade MeOH and incubate the mixture on ice for at least 5 min.
24. Centrifuge the sample at 15,300 × *g* for 10 min at 4°C and collect the supernatant.
25. Dilute the supernatant 10-fold and inject 3–6 μL of the supernatant into an appropriate UPLC-ESI-qTOF instrument equipped with a C18 reverse-phase UPLC column.

***Note:*** Thn is more susceptible to acid-mediated hydrolysis. To observe the Thn-containing precursor peptides, an H_2_O/MeCN mobile phase containing 0.1% acetic acid is recommended for LC-MS, instead of 0.1% formic acid to avoid hydrolysis of Thn.

## Expected outcomes

**Figure 3:**
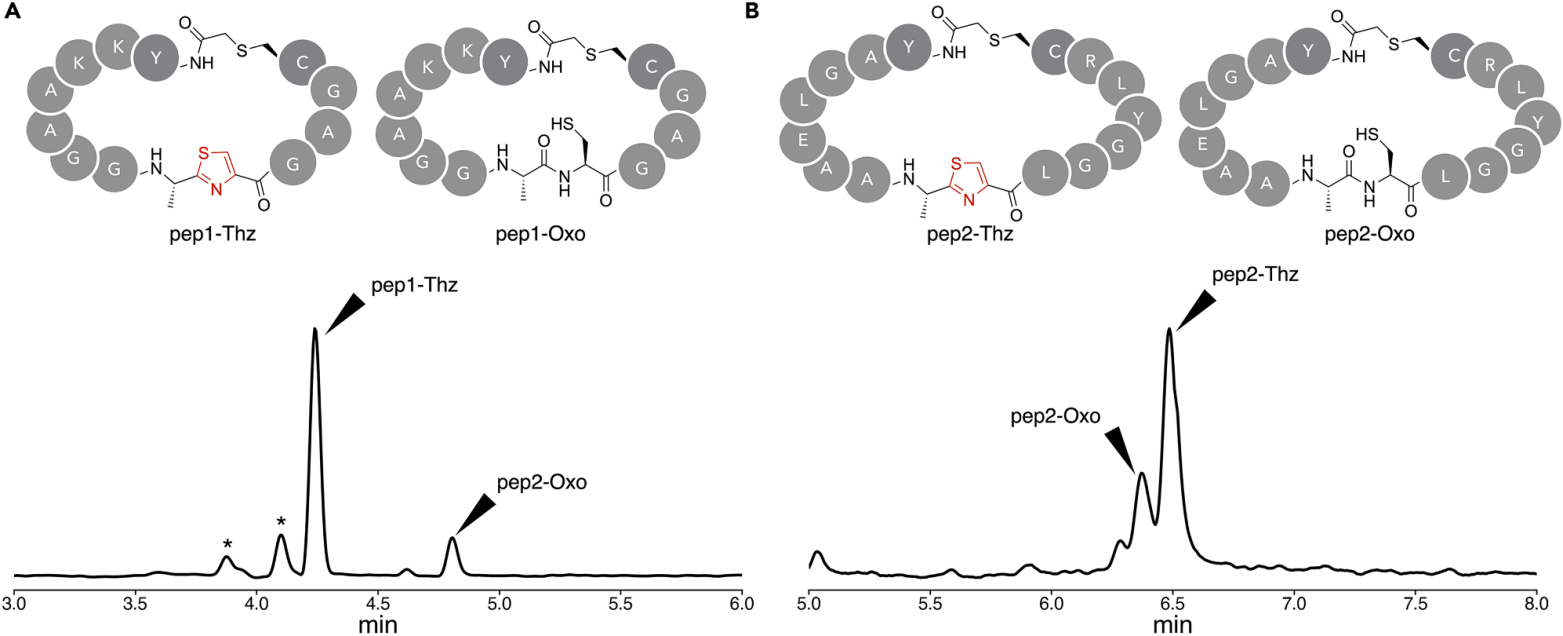
Representative LC-MS analysis of ^Thz^teMP synthesis (A) An extracted ion chromatogram for pep1-Thz (m/z 588.7504) and pep1-Oxo (m/z 598.7635). Asterisks indicate disulfide adducts with thiols at the Cys residue. (B) An extracted ion chromatogram for pep2-Thz (m/z 825.8743) and pep2-Oxo (m/z 835.8874).

LC-MS results typically detect the desired ^Thz^teMPs along with oxoamide-containing byproducts exhibiting a +20 Da mass shift relative to the ^Thz^teMPs (Figure 3). Under the optimized conditions described here, the ^Thz^teMP products generally constitute approximately 70–80% of the total products detected by LC-MS. As examples, we show the results for two representative ^Thz^teMP sequences (pep1-Thz, pep2-Thz). Depending on the reaction conditions, some peptide sequences may produce the oxoamide byproducts partially as thiol adducts at the Cys residue (Figure 3A), while others do not (Figure 3B). These representative spectra serve as references for interpreting the LC-MS results obtained from other ^Thz^teMP sequences.

## Limitations

Although this protocol provides a versatile method to access various ^Thz^teMPs, several limitations should be considered. First, a major limitation arises from the *S-to-O* conversion of Ala^S^-tRNA. Even under carefully controlled conditions, this side reaction inevitably occurs in aqueous solution, resulting in the concomitant formation of corresponding oxoamide byproducts alongside the desired ^Thz^teMPs. In particular, the installation of multiple Thz moieties, especially at successive positions, within a single peptide chain is expected to be relatively inefficient, which should be taken into consideration when designing highly modified peptide libraries for screening applications. In addition, hydrolysis of aminoacyl-tRNA represents another potential side reaction that can reduce the yield of the desired products, especially under prolonged handling times or suboptimal conditions. The protocol described here has been optimized to minimize these side reactions; therefore, strict adherence to the procedure is important.

At present, Thz formation has been demonstrated only for an Ala-type thioamide substrate. In principle, however, other Thz side chains should also be accessible by preparing the corresponding amino(thio)acid substrates. Such modification will likely require re-optimization of both the flexizyme-mediated aminoacylation conditions and the subsequent *in vitro* reprogrammed translation conditions.

Although GodE exhibits relatively broad substrate tolerance, its activity is particularly influenced by the local amino acid composition surrounding the modification site. For example, bulky or charged residues upstream of the Thn site often reduce the efficiency of enzymatic oxidation to Thz. Therefore, the sequence surrounding the Thz moiety in the target ^Thz^teMP should be designed carefully. In particular, residues immediately downstream of the Thz site are generally preferred to be small and uncharged amino acids, such as Gly, Ala, Ser, Thr, and Pro.^1^

## Troubleshooting

### Problem 1

A sufficient amount of the desired product cannot be isolated by column chromatography (related to steps 2 and 6).

### Potential solution

Consider using fresh anhydrous DCM. Additionally, drying DIPEA over molecular sieves (4 Å) prior to use may improve product recovery. Ensure the reaction is performed under an inert atmosphere (using either nitrogen or argon).

### Problem 2

The deprotected product contains a large amount of *S-to-O*-converted Ala-DNB in MS analysis (related to step 14).

### Potential solution

Use freshly purchased 4 N HCl in EtOAc to minimize moisture contamination, which triggers the conversion. Moisture contamination in other reagents should also be minimized, and old reagents should be replaced with fresh ones whenever possible.

### Problem 3

*S-to-O* converted byproducts account for more than 50% of the products detected by LC-MS (related to step 20).

### Potential solution

Make sure to wash the aminoacyl-tRNA pellets thoroughly in step 16 to avoid carry-over of residual Mg^2+^, which can significantly suppress the translation efficiency. If the problem persists, *S-to-O* conversion may have proceeded during storage due to moisture contamination. Check the MS spectrum of the amino(thio)acid substrate and evaluate the ratio of Ala^S^-DNB to Ala-DNB. If the Ala^S^-DNB solution is of sufficient quality, the final concentration of Ala^S^-DNB in the aminoacylation reaction should be optimized within the range of 5–40 mM to improve Ala^S^ incorporation efficiency.

### Problem 4

Significant amounts of oxoamide byproduct appear as Cys or β-mercaptoethanol adducts (related to step 22).

### Potential solution

Adding a reducing agent such as tris(2-carboxyethyl)phosphine (TCEP) or an alkylating agent such as iodoacetamide (IAA) after enzymatic oxidation may simplify LC-MS analysis of the byproducts.

### Problem 5

Low conversion efficiency of GodE. (related to step 22).

### Potential solution

Ensure the GodE stock solution has not undergone repeated freeze–thaw cycles. Aliquot the enzyme upon first use to maintain activity. As mentioned in limitations section, redesigning the sequence surrounding the modification site may improve conversion efficiency.

## Resource availability

### Lead contact

Further information and requests for resources and reagents should be directed to and will be fulfilled by the lead contact, Yuki Goto (goto.yuki.4x@kyoto-u.ac.jp).

### Technical contact

Technical questions on executing this protocol should be directed to and will be answered by the technical contacts, Akihiro Saito (saito.akihiro.88r@st.kyoto-u.ac.jp).

### Materials availability

This study did not generate new unique reagents.

### Data and code availability

The datasets supporting the current study are available within the article.

## Acknowledgments

We thank Prof. H. Suga, Prof. H. Onaka, and Dr. H. Kimura for their contributions to the development of the original methodology underlying this protocol. This work was partially supported by KAKENHI (JP24K01634, JP24H01754, JP24K21760, and JP25H01578) from the 10.13039/501100001691Japan Society for the Promotion of Science and Adopting Sustainable Partnerships for Innovative Research Ecosystem (JPMJAP2418) from 10.13039/501100002241Japan Science and Technology Agency.

## Author contributions

Y.G. supervised the original study and this manuscript. A.S. performed the experimental work. Y.G. and A.S. wrote the manuscript.

## Declaration of interests

The authors declare no competing interests.
